# Four-Week vs Six-Week Antibiotic Therapy in the Management of Nonsurgically Treated Diabetic Foot Osteomyelitis: Protocol for a Multicentric, Single-Blind Randomized Clinical Trial

**DOI:** 10.2196/93492

**Published:** 2026-06-26

**Authors:** Amritava Ghosh, Ashu Rastogi, Pranab Kumar Sahana, Alok Chandra Agrawal, Amit Kumar Mishra, Debajyoti Mohanty, Narendra Kuber Bodhey, Pragya Agarwala, Rachita Nanda, Mukul Bhattacharyya, Raja Ray, Archana Singh, Soumik Das, Mousumi Mukhopadhyay, Dibyendu Biswas

**Affiliations:** 1 Department of Endocrinology & Metabolism All India Institute of Medical Sciences Raipur Raipur, Chhattisgarh India; 2 Post Graduate Institute of Medical Education and Research Chandigarh, Chandigarh India; 3 Institute of Post Graduate Medical Education and Research Kolkata, West Bengal India; 4 All India Institute of Medical Sciences Raipur Raipur, Chhattisgarh India

**Keywords:** diabetes mellitus, diabetic foot, infection, osteomyelitis, antibiotics

## Abstract

**Background:**

Diabetic foot osteomyelitis (DFO) is a common complication and major cause of morbidity among people with diabetes mellitus. There has been growing acceptance of primarily nonsurgical (conservative) management of DFO based on antibiotics alone. However, the most appropriate duration of antibiotic therapy for DFO remains controversial. Current guidelines recommend antibiotic duration of up to 6 weeks for DFO. Although there has been growing interest in a shorter duration of antibiotic therapy, in absence of sufficient evidence, the extent to which the duration of antibiotic therapy can be shortened remains debatable. Determination of the optimal duration of antibiotic therapy would improve the outcomes of treatment of DFO while limiting side effects.

**Objective:**

This study aims to determine the rates of remission (primary objective) and adverse events (secondary objective) associated with 4 vs 6 weeks of antibiotic therapy in nonsurgically treated DFO.

**Methods:**

In this single-blind randomized clinical trial, eligible people with DFO who give consent will be randomized to 4 or 6 weeks of antibiotic therapy in a 1:1 ratio. Participants will be treated according to current guidelines. First, an empirical antibiotic therapy to be modified if required according to the results of bone culture will be administered. Additional supportive therapy (wound care, pressure off-loading, and professional care for control of glucose levels and other comorbidities) will be provided as indicated. Participants will be followed up until 6 months after antibiotic discontinuation. Radiographs and magnetic resonance images will be obtained at baseline, and at 6 weeks and after 6 months of discontinuation of antibiotics. Hematologic, renal, and hepatic parameters will be assessed during treatment. DFO remission—defined as a composite of sustained wound healing, absence of local or systemic signs of infection, stabilization or improvement in radiologic signs, absence of recurrence of osteomyelitis at the initial or contiguous site, and absence of requirement of amputation or bone resection—will be prospectively assessed at 6 months after end of antibiotic therapy. The investigator responsible for the outcome assessment will be blinded to participants’ group assignment. The sample size was calculated to be 52 participants in each group. The randomized clinical trial will be a noninferiority study. Outcomes will be published as intention-to-treat and per-protocol database.

**Results:**

This study received sanction of research grant and budget allotment in March 2025. Data collection commenced in December 2025. As of February 2026, a total of 7 participants have been enrolled. The results are expected to be published by the first quarter of 2029.

**Conclusions:**

This study is expected to generate high-quality evidence on the optimum duration of antibiotic therapy in DFO.

**Trial Registration:**

Clinical Trials Registry - India CTRI/2025/02/080469; https://tinyurl.com/2aef5ex7

**International Registered Report Identifier (IRRID):**

DERR1-10.2196/93492

## Introduction

Diabetic foot osteomyelitis (DFO) is a common complication and major cause of morbidity among people with diabetes mellitus. The prevalence of osteomyelitis in diabetic foot infection (DFI) ranges from approximately 20% in mild DFI to up to 60% in severe DFI, requiring hospitalization [1].

The current practices for treatment of DFO vary widely, likely due to a lack of convincing data to guide therapeutic practice. While, traditionally, treatment of DFO has involved surgery, including excision of infected bone or amputation, there has been growing interest in primarily nonsurgical (conservative) management based on antibiotics alone [2]. Predominantly antibiotic therapy without ablative surgery is often effective, with reported remission rates ranging from 53% to 82% [3-13]. It also offers a number of other advantages, such as avoidance of surgery and its associated complications and morbidity, preserved biomechanics of the foot with resultant reduction in risk of reulceration, reduced need for hospitalization, and cost reduction.

An important area of controversy in nonsurgical management of DFO is the duration of antibiotic therapy. The optimum duration of antibiotic therapy in nonsurgically treated DFO is currently unknown. A major contributor to this controversy is the absence of an established clinical, biological, or radiological factor predictive of remission that might help physicians decide when to discontinue treatment. While several markers have been suggested (such as resolution of soft tissue infection, healing of the wound, reduction in levels of inflammatory markers, and improvement in radiographic changes), there is absence of strong evidence in the literature to support their use [1]. In absence of any reliable clinical, biological, or radiological marker, the currently used definition of remission is the absence of persistent or new-onset DFO at the initial or contiguous site for a minimum of 6 months after completion of antibiotic therapy [14].

In most of the previously published literature, the duration of antibiotic therapy has been more than 12 weeks for some or all the participants [3,4,6-11]. Two small randomized controlled studies reported similar rates of recovery with 6 weeks compared to 12 weeks of antibiotic therapy [13,15]. The International Working Group on the Diabetic Foot and Infectious Diseases Society of America 2023 guidelines on the diagnosis and treatment of diabetes-related foot infections have recommended antibiotic duration of up to 6 weeks for DFO in absence of bone resection or amputation, although no lower limit has been proposed [14]. In comparison, other publications have recommended a therapy duration of 4 to 6 weeks for DFO [16-19], although the data supporting this are limited. Revascularization of the infected bone takes 3 to 4 weeks [20]. Recently, there has been growing interest in even shorter durations of antibiotic therapy for the treatment of DFO. While limited literature suggests 3 weeks of antibiotics to be noninferior to 6 weeks for treatment of residual bone infection following partial surgical treatment of DFO [21,22], data on shorter duration of antibiotic therapy in nonsurgically treated DFO are limited. In the absence of sufficient evidence, the extent to which the duration of antibiotic therapy can be shortened remains debatable, and the optimal duration of antibiotic treatment in DFO continues to be an area of uncertainty [14]. Long-term use of antibiotics incurs financial costs, may be associated with drug-related adverse effects, and also encourages antimicrobial resistance. A shorter duration of antibiotic therapy may help mitigate these drawbacks. Therefore, studies evaluating shorter duration of antibiotic therapy in nonsurgically treated DFO are necessary.

This study aims to compare the rate of remission (primary objective) and type and rate of adverse events (secondary objective) of 4 vs 6 weeks of antibiotic therapy in the management of nonsurgically treated DFO.

## Methods

### Trial Design

This single-blind multicenter randomized clinical trial will be conducted in 3 tertiary care centers across India over a period of 46 months. Consecutive people with DFO seen in an outpatient setting or during hospitalization in the department of endocrinology and metabolism or the department of general surgery at the participating centers will be considered for the study. Eligible people will be informed about the study and invited to participate. People who do not consent to be included in the study will be excluded. The study will include adults with forefoot DFO who are willing to undergo a follow-up of 6 months after completion of antibiotic treatment and accept local wound care and off-loading. Pregnant or lactating women and those with peripheral arterial disease, gangrene, indication for bone resection or amputation, material-related infection, or an estimated glomerular filtration rate of less than 30 mL/min/1.73 m^2^ will be excluded. Additionally, those with any concurrent infection requiring systemic antibiotic therapy for more than 10 days or those who have received potentially effective systemic antibiotic therapy for more than 5 days with the wound showing clinical improvement will be excluded

### Ethical Considerations

Confidentiality of the data collected will be maintained. The protocol of the study has been approved by the ethics committees of the participating institutes (letters 4883/IEC-AIIMSRPR/2025 and PGI/IEC/2025/EIC000167 and memo IPGME&R/IEC/2025/0053) and will be performed in accordance with the principles of the current version of the Declaration of Helsinki, good clinical practice standards, and the Indian Council of Medical Research ethical guidelines. The study is registered with the Clinical Trials Registry - India with registration number CTRI/2025/02/080469. Only people who give written informed consent will be included in the study and evaluated as per the protocol.

### Characteristics of the Groups and Interventions

Participants will be allocated to 1 of the following 2 groups: 4-week antibiotic therapy arm or 6-week antibiotic therapy arm.

### Randomization and Group Allocation

The single-blinded allocation to the 4-week or 6-week antibiotic therapy arm will be conducted electronically in a 1:1 ratio (randomization without blocked or matched variables). The random numbers for allocation of study participants will be generated at the central study site using freely accessible randomization programs. The information regarding allocation to 1 of the 2 groups will be kept inside opaque, sealed envelopes. The participants will be allocated to the 4- or 6-week antibiotic therapy arm based on the sequence in which they enter the study (ie, the sealed envelope bearing the corresponding sequence will be opened by a member of the study team at the coordinating study site, and the information about group allocation will be shared with the study site where the participant will be recruited).

### Methodology

Detailed history (including symptoms and information on diabetes and chronic complications), findings of examinations (including characteristics of the diabetic foot ulcer), laboratory parameters, and radiological findings will be recorded in the case record form (Multimedia Appendix 1). Participants will be screened for evidence of peripheral neuropathy using monofilament (10 g) and a biothesiometer. Vascular assessment will be performed at baseline via arterial doppler and measurement of ankle brachial index with or without toe systolic pressure. A probe-to-bone test will be conducted on all participants. All participants will have radiographs and magnetic resonance imaging (MRI) centered on the site of infection. Bone biopsy specimens will be obtained for aerobic as well as anaerobic cultures. Ulcers will be classified according to the University of Texas ulcer classification system [23] and the perfusion, extent, depth, infection, and sensation classification system [24].

Individuals will be treated according to current therapeutic practices. First, an empirical antibiotic therapy to be modified if required according to the results of culture and sensitivity tests when they become available will be administered. The choice of agent will be as per the preference of the treating clinicians guided by the International Working Group on the Diabetic Foot and Infectious Diseases Society of America 2023 guidelines and considering allergies and comorbidities. Nonetheless, a list of “allowed antibiotics” has been established to maintain minimal uniformity (Tables 1 and 2). Topical antibiotics and antiseptics will be avoided. Antibiotics will be administered either orally for the entire duration of therapy or, if required, intravenously for an initial period of 5 to 7 days followed by orally for the remainder of the therapy duration. During outpatient therapy, participants will be instructed to return the empty packages of antibiotics, which will serve as a surrogate proof of intake.

All participants will receive standard diabetic foot wound care in the form of debridement (if clinically indicated during hospitalization and outpatient visits), daily dressing changes, and pressure off-loading and professional care for control of glucose levels and other comorbidities [25]. Because off-loading is critical to the healing process, strategies for off-loading will be standardized. All participants with ulcers in the plantar aspect of the foot will be equipped with a suitable off-loading device (removable cast walker) during baseline visit 1 once the lesion has been debrided and cleansed and an appropriate dressing has been applied. The size of the removable cast walker will be ascertained based on the participant’s correct shoe size. An insole of appropriate size will be inserted into the walker, and the device will be applied according to instructions provided by the manufacturer. Participants will be instructed to wear the off-loading device at all times, including during night rest. They will be switched to customized footwear after healing of the ulcer.

**Figure 1:**
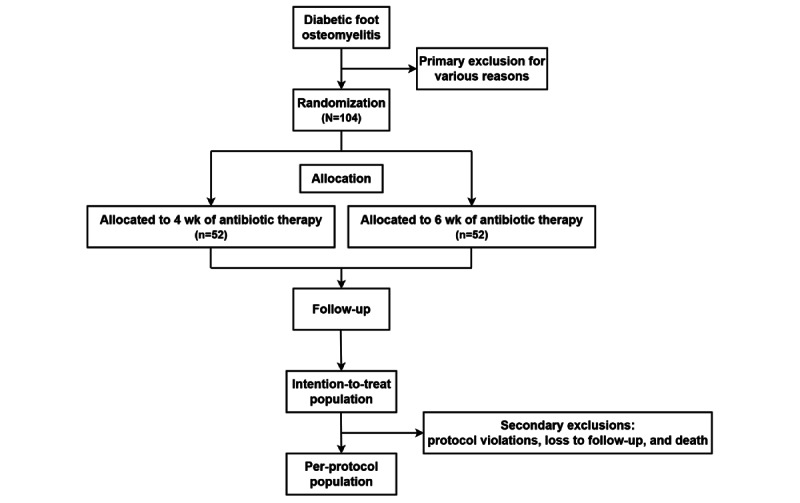
Flowchart of the research methodology.

Participants will be followed up once every 2 weeks for 6 weeks or until healing of the ulcer, whichever is later, and then once every 2 months until relapse (Textbox 1) or for at least 6 months after antibiotic discontinuation, whichever is earlier [13,14,25-27]. The assessments will be based on the study objectives (primary and secondary outcomes). Participants will also be advised to report in between scheduled visits when ulcers heal and also if they observe any deterioration in the condition of the foot. Participants will also be observed for development of any new ulcers or recurrence of ulcers that had healed. Follow-up radiographs and MRIs will be obtained at 6 weeks and after 6 months of antibiotic discontinuation. Renal, hepatic, and hematologic parameters will be monitored during treatment. DFO remission will be prospectively assessed at 6 months after the completion of antibiotic therapy. Appropriate medical and/or surgical treatment will be initiated in the event of a relapse. Only 1 episode of DFO will be considered for the study in a given participant. The study-related information, clinical assessments, and laboratory tests to be conducted at each visit of the study participants have been summarized in Table 3. The research methodology has been summarized in the form of a flowchart in Figure 1.

**Table 1:** List of the allowed antibiotic treatments (empirical or targeted).

Antibiotic agent	Allowed dosing regimens	Allowed daily total range
Levofloxacin PO^a^	750 mg every 24 h or 500 mg every 12 h	750-1000 mg
Ciprofloxacin PO	750 mg every 24 h or 500 mg every 12 h	750-1000 mg
Amoxicillin and clavulanate PO	500 and 125 mg every 12 or 8 h	1000 and 250 mg to 1500 and 375 mg
Amoxicillin and clavulanate IV^b^	1000 and 200 mg every 12 or 8 h	2000 and 400 mg to 3000 and 600 mg
Cefuroxime PO	500 mg every 12 h	1000 mg
Cefuroxime IV	1500 mg every 8 h	4500 mg
Ceftriaxone IV	2000 mg every 24 h	2000 mg
Cotrimoxazole PO	960 mg every 12 or 8 h	1920-2880 mg
Clindamycin PO	300 or 450 mg every 6 h	1200-1800 mg
Doxycycline PO	100 mg every 12 h	200 mg
Linezolid PO	600 mg every 12 h	1200 mg
Linezolid IV	600 mg every 12 h	1200 mg
Metronidazole PO	400 mg every 8 h or 6 h	1200-1600 mg
Metronidazole IV	500 mg every 8 or 6 h	1500-2000 mg
Vancomycin IV	15 mg/kg every 12 h	According to serum levels (10-20 mg/L)
Meropenem IV	1 or 2 g every 12 or 8 h	2-6 g
Piperacillin and tazobactam IV	4000 and 500 mg every 8 h	12000 and 1500 mg (12 and 1.5 g)

^a^PO: oral therapy.

^b^IV: intravenous therapy.

**Table 2:** Guidance for selecting an empirical antibiotic regimen for 3O (moderate infection involving bone or osteomyelitis) or 4O (severe infection involving bone or osteomyelitis) (adapted from Senneville et al [14])^a^.

Infection severity and additional factors	Usual pathogens	Potential empirical regimens
No complicating features	GPC^b^ with or without GNR^c^	Amoxicillin and clavulanate Cefuroxime Ceftriaxone
Recent antibiotics	GPC with or without GNR	Piperacillin and tazobactam Cefuroxime Ceftriaxone
Macerated ulcer or warm climate	GNR, including *Pseudomonas*	Piperacillin and tazobactam Meropenem
Ischemic limb, necrosis, or gas forming	GPC with or without GNR with or without anaerobes	Amoxicillin and clavulanate Piperacillin and tazobactam Meropenem Cefuroxime or ceftriaxone+clindamycin or metronidazole
MRSA^d^ risk factors	MRSA	Consider adding or substituting with vancomycin, linezolid, trimethoprim and sulfamethoxazole, or doxycycline
Risk factors for resistant GNR	ESBL^e^	Meropenem FQ^f^ Aminoglycoside Colistin

^a^Where multiple antibiotics have been listed, only 1 should be selected unless otherwise indicated. For individuals with concomitant conditions such as renal or hepatic dysfunction or obesity, modification of antibiotics or doses should be considered. Parenteral antibiotics should initially be preferred for severe infections, with switch to oral antibiotics as follow-up.

^b^GPC: gram-positive cocci (staphylococci and streptococci).

^c^GNR: gram-negative rod.

^d^MRSA: methicillin-resistant *Staphylococcus aureus*.

^e^ESBL: extended-spectrum β-lactamase–producing organism.

^f^FQ: fluoroquinolone with good activity against aerobic GPC (eg, levofloxacin).

**Operational definitions.**

- Diagnosis of diabetes mellitus will be as per the American Diabetes Association Standards of Care in Diabetes 2024 [26].
- Diabetic foot osteomyelitis (DFO) will be defined by the association of soft tissue infection and/or positive probe-to-bone test, with radiologic signs indicative of osteomyelitis on plain radiographs (disruption of cortex, periosteal elevation, involucrum, sequestrum, or gross bone destruction) and/or magnetic resonance imaging (bone marrow edema, periostitis, intraosseous or subperiosteal abscess, sinus tract, cloaca, sequestrum, or involucrum) [25].
- Forefoot will be defined as the anterior part of the foot, consisting of phalanges, metatarsals, and associated soft tissues. However, participants with more proximal infections will be eligible as long as these originate in the forefoot.
- Peripheral neuropathy will be defined as loss of protective sensation on testing using a 10-g monofilament along with impairment of one of the following: pinprick sensation, vibration sense using a 128-Hz tuning fork, vibration perception threshold, or ankle reflexes.
- Peripheral arterial disease will be defined as ankle brachial index<0.9 and/or a toe systolic pressure<70 mm Hg.
- Diabetic retinopathy will be defined according to the International Classification of Diabetic Retinopathy and Diabetic Macular Edema [27]. Accordingly, the presence of any of the features of nonproliferative diabetic retinopathy (mild, moderate, or severe), proliferative diabetic retinopathy, or diabetic macular edema (mild, moderate, or severe) will be considered as presence of diabetic retinopathy.
- Diabetic kidney disease will be defined as an estimated glomerular filtration rate of <60 mL/min/1.73 m^2^ and/or a urinary albumin-to-creatinine ratio of ≥30 mg/g of creatinine.
- Hypertension will be defined as a known history of hypertension with specific drug treatment or blood pressure of ≥140/90 mm Hg.
- Remission of DFO will be defined as a composite of (1) complete and sustained healing of the wound resulting in osteomyelitis, (2) absence of local or systemic signs of infection, (3) stabilization of or improvement in radiologic signs of osteomyelitis, (4) absence of recurrence of osteomyelitis at the initial or contiguous site, and (5) absence of requirement of amputation or surgical bone resection assessed at 6 mo after the end of antibiotic therapy [13,14].
- Failure will be defined as any other outcome.
- Relapse will be defined as recurrent bone infection at the initial site.
- Healing of ulcer will be defined as skin closure.
- Ulcer healing time will be calculated starting on the day of initiation of antibiotic therapy.
- The imputability of adverse events related to antibiotic treatment will be determined based on the type of documented toxicity; the time of onset of the event; the necessity of discontinuation or reduction in dose of the implicated antibiotic; and the effect of the attempt, if any, to reintroduce the antibiotic. To be attributable to a particular antibiotic, the need for discontinuation or reduction in dose of the antibiotic due to intolerance should have been documented in the participant’s medical record.

**Table 3:** Details of the study-related information, clinical assessment, and laboratory tests planned to be obtained at different visits of the study participants.

	Baseline visit 1—day 0	Visit 2—day 14	Visit 3—day 28	Visit 4; EOT^a^—day 42	Visits 5 and 6—2 and 4 mo after EOT	Visit 7; test of cure—6 mo after EOT
Radiograph, MRI^b^, ESR^c^, and CRP^d^	✓			✓		✓
Inclusion and exclusion criteria	✓					
Informed consent	✓					
Demographics	✓					
Medical history	✓					✓
Neuropathy and vascular assessment	✓					
Clinical assessment of infection	✓	✓	✓	✓	✓	✓
Culture and sensitivity tests	✓					
Control of compliance		✓	✓	✓		
Adverse events		✓	✓	✓		
Renal, hepatic, and hematologic parameters	✓		✓	✓		

^a^EOT: end of trial.

^b^MRI: magnetic resonance imaging.

^c^ESR: erythrocyte sedimentation rate.

^d^CRP: C-reactive protein.

### Withdrawal Criteria

To safeguard the best interests of the participants, the researchers may terminate a person’s participation in case of development of any indications for bone resection or amputation.

### Outcome Measures

The primary outcome measure will be remission of DFO (Textbox 1), whereas the secondary outcome measure will be adverse events associated with antibiotic therapy.

### Blinding

The investigator at each site responsible for outcome assessment will be blinded to group assignment and prescriptions offered to the participants. An assessor blinded to randomization will be responsible for evaluation of the participants at each visit for status of the ulcer, signs of infection, and wound healing progress. The assessor will also evaluate the participants for any adverse events and any signs of recurrence of osteomyelitis. Outcome will be determined based on predefined criteria as per the operational definitions (Textbox 1).

### Sample Size

The sample size has been estimated to be 46 participants in each group (4- and 6-week treatment arms) using the nMaster software (version 2; Biostatistics Resource and Training Centre) with a 95% level of confidence; 80% power; and a 10% noninferiority margin considering the remission of DFO among 3- and 6-week treatment groups of 84% and 73%, respectively, in a previous study [21]. We estimate that 10% of study participants may later withdraw consent or be lost to follow-up. Therefore, the final sample size with a 10% nonresponse rate has been calculated as 52 participants in each group.

### Statistical Analysis

The collected data from the study participants as per the objective will be entered into Microsoft Excel. The required data will be compiled from all the study sites and checked for completeness as per the objectives. Data cleaning will be conducted before proceeding with data analysis. Analysis of compiled data will be conducted using SPSS Statistics (version 29.0.2.0; IBM Corp) for Windows. Data as per the study objectives will be presented either in tabular or graphical form.

The randomized clinical trial will be a noninferiority study without an adaptive study design. The primary outcome measures are binary variables. The categorical data will be presented as frequencies, percentages, and proportions. Quantitative data will be checked for normal distribution (Shapiro-Wilk test) and presented either as means and SDs or medians and IQRs based on the type of distribution (ie, normal or nonnormal, respectively). The application of parametric or nonparametric tests to compare the quantitative variables between the 2 groups will be based on the distribution of the quantitative variables (normal or nonnormal, respectively). Statistical tests such as the independent *t* test (2-tailed) or Mann-Whitney *U* test will be used to compare the quantitative variables between the groups. Chi-square tests will be conducted to compare the distribution of categorical variables between the groups.

The risk for outcome variables will be measured using the relative risk or risk ratio. The crude risk will be assessed via univariate analysis, and the independent variables or factors on univariate analysis with a *P* value of less than .20 will be considered for multivariate regression analysis to assess the adjusted risk ratio. Cox regression analysis will be conducted for time-to-event data for outcomes such as remission or any adverse event following antibiotic therapy. For statistical tests, statistical significance will be established when the *P* value is less than .05. Intention-to-treat and per-protocol analyses will be conducted. Intention-to-treat analysis will give an idea of the real-world situation considering the data of all participants irrespective of their duration of participation, and per-protocol analysis will give an idea of the actual effectiveness of the intervention as compared to the control group considering the participants who complete the study as per the protocol.

**Figure 2:**
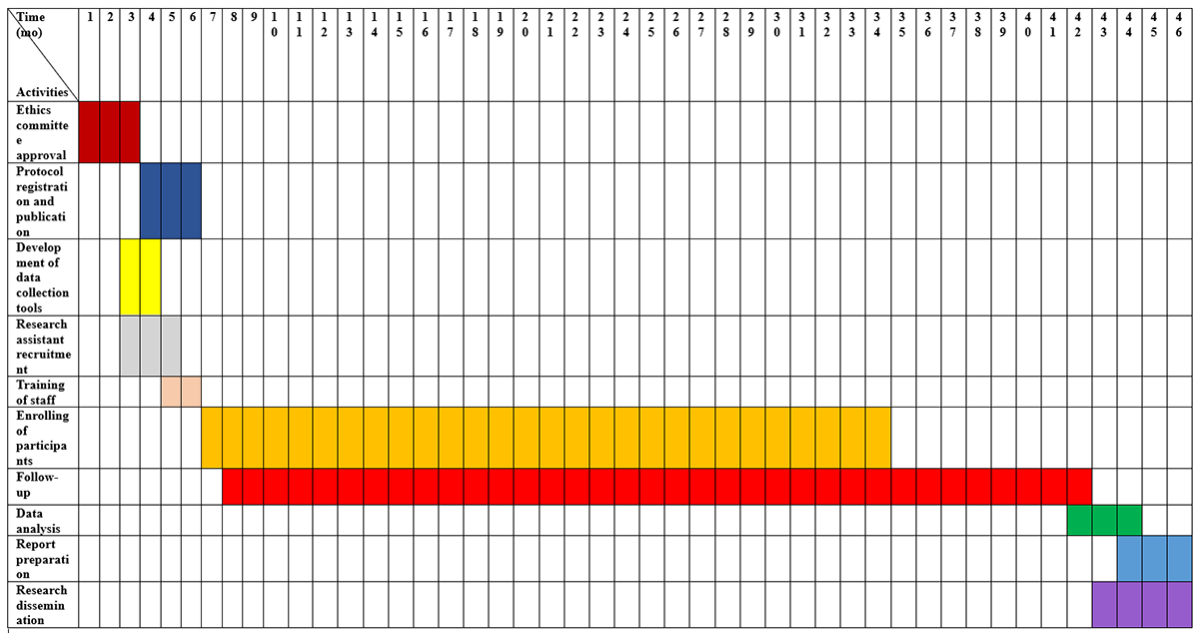
Timelines with achievable targets.

The timeline of the research project along with achievable targets are shown in Figure 2.

## Results

The study received sanction of research grant and budget allotment in March 2025. Data collection commenced in December 2025. As of February 2026, a total of 7 participants have been enrolled. The study is expected to be completed by 2028, and the results are expected to be published by the first quarter of 2029.

## Discussion

In this study, the rates of remission and rates and types of adverse events associated with 4 and 6 weeks of antibiotic therapy for nonsurgically treated DFO will be determined. Therefore, this study will provide an indication of whether a shorter duration of antibiotic therapy of 4 weeks is as effective as and safer than the currently recommended duration of 6 weeks. In addition, the study is expected to generate high-quality data on clinico-microbiological profiles of DFO from 3 tertiary care centers across India.

A nonsurgical (conservative) approach that relies primarily on antibiotic therapy is often effective in the treatment of DFO. The success of antibiotic therapy for DFO is influenced by multiple factors, such as the choice of antibiotic, the susceptibility of the bacteria located in the bone to the administered agent, the vascular status of the affected limb, and other systemic factors such as renal function. The duration of antibiotic therapy is also a key determinant of success of medical treatment. However, the optimum duration of antibiotic treatment for DFO remains controversial.

Previous studies exploring nonsurgical management of DFO have been retrospective in design. These studies have reported prolonged antibiotic therapy, with the median duration ranging from 11 weeks to 6 months. Remission was achieved in 64% to 81% of participants [4,8,10,28]. In a prospective randomized trial, similar rates of remission (60% vs 70%, respectively; *P*=.50) with fewer antimicrobial therapy–related adverse events (15% vs 45%, respectively; *P*=.04) were reported in those receiving antibiotic therapy for 6 weeks compared to those receiving it for 12 weeks [13]. Another prospective interventional study also reported similar rates of recovery with 6 vs 12 weeks of antibiotic therapy (78.6% vs 84.6%; *P*=.74) [15]. In a recent pilot randomized controlled trial among participants with DFO, 3-week antibiotic therapy after debridement was associated with similar (statistically noninferior) rates of remission and adverse effects compared with 6-week antibiotic therapy. However, it should be noted that, in this trial, the duration of follow-up after end of therapy was only 2 months [21]. All the studies conducted to date have had limitations of small sample sizes.

Thus, very few studies have prospectively evaluated the outcomes associated with various durations of antibiotic treatment. While long-term antibiotic treatment has traditionally been used, there has been growing interest in shorter durations of antibiotic therapy in nonsurgically treated DFO. However, owing to a paucity of large randomized controlled trials, the most appropriate duration of antibiotic treatment for the management of DFO is not known. Although current guidelines recommend antibiotic duration of up to 6 weeks for treatment of DFO, the recommendations are only conditional and based on low levels of evidence. Additional research is necessary to determine the appropriate duration of antibiotic treatment for DFO. Long-term use of antibiotics incurs financial costs, may be associated with drug-related adverse effects, and also encourages antimicrobial resistance. Hence, it is important to evaluate shorter treatment durations of antibiotics to reduce the costs and complications associated with them and the emerging threat of antimicrobial resistance. However, so far, the efficacy of an antibiotic course of less than 6 weeks has been evaluated in only 1 randomized controlled trial, which had major limitations of small sample size and short follow-up duration after end of treatment. In the absence of adequate evidence of efficacy, the extent to which the currently recommended durations of antibiotic therapy for DFO can be shortened remains controversial. Determination of the optimal duration of antibiotic therapy for DFO would improve outcomes of treatment while limiting side effects. This study aims to compare the effectiveness and tolerance of 4 vs 6 weeks of antibiotic therapy in the management of nonsurgically treated DFO.

The strength of this protocol is its randomized, single-blind, 2–parallel arm controlled study design and large sample size. The use of MRI, which has high diagnostic accuracy for DFO [29], in the initial evaluation and follow-up of participants and of bone biopsy with aerobic and anaerobic culture to guide antibiotic therapy further adds to the strength of the study. Uniform and standardized methods of off-loading will be followed for all participants. The precise operational definitions of DFO, remission, failure, relapse, healing of ulcer, ulcer healing time, and imputability of adverse events make the protocol robust. The clearly defined outcome measures (Textbox 1) and outcome assessment by investigators blinded to group assignment and prescriptions offered to the participants are expected to reduce bias. Additional strengths of the study include a long follow-up period and a multicenter study design. We intend to recruit participants from multiple tertiary care centers from across the nation, each having advanced foot care facilities.

A large case mix of moderate to severe infection and a variety of microorganisms having varying degrees of sensitivity to antimicrobials are expected among the study participants. Additionally, prevalent participant characteristics may preclude the use of certain antibiotics. Hence, the choice of antibiotic and route of administration have not been prespecified. However, treatment guided by aerobic and anaerobic bone culture and sensitivity results will ensure an appropriate choice of effective antibiotics. Nevertheless, to achieve minimal homogeneity, a list of “allowed antibiotics” has been established, as detailed in Tables 1 and 2. Moreover, the process of randomization of participants to 1 of the 2 groups is expected to minimize bias and ensure that the study groups are comparable. To add to that, the selection of antibiotic and its route of administration depending on culture results and clinician preference is expected to provide an unbiased, real-world estimate of treatment effectiveness.

Following project completion, steps will be taken to disseminate the results of the study through participation and presentation in scientific conferences and publication in scientific journals. The results of this study may pave the way for further larger studies evaluating shorter durations of antibiotic therapy for the management of DFO.

In conclusion, while there is growing acceptance of primarily nonsurgical (conservative) management of DFO based on antibiotics alone, the duration of antibiotic therapy for DFO remains controversial. Although current guidelines recommend antibiotic duration of up to 6 weeks for DFO, there has been growing interest in shorter durations of antibiotic therapy. This study is expected to offer much needed information regarding the optimum duration of antibiotic therapy for the management of DFO and pave the way for better outcomes of predominantly nonsurgically managed DFO while minimizing financial burden, drug-related adverse effects, and antimicrobial resistance.

## Appendix

Multimedia Appendix 1 Data collection tool and study checklist.

## Abbreviations

DFI: diabetic foot infection
DFO: diabetic foot osteomyelitis
MRI: magnetic resonance imaging
