# Supplementary material for: Four-Week vs Six-Week Antibiotic Therapy in the Management of Nonsurgically Treated Diabetic Foot Osteomyelitis: Protocol for a Multicentric, Single-Blind Randomized Clinical Trial
Source: JMIR Res Protoc. 2026 Jun 26;15:e93492. doi: 10.2196/93492 (PMC13354949; doi:10.2196/93492)
Supplement: Multimedia Appendix 1 [file resprot_v15i1e93492_app1.docx]

**Case Record Form**

**Title of Project:** Single blind randomized clinical trial on 4 weeks versus 6 weeks antibiotic therapy in the management of non-surgically treated diabetic foot osteomyelitis.

# Visit 1 — Baseline (Day 0)

1. **Inclusion and Exclusion Criteria:**

**1.1 Inclusion Criteria**

| **S. No.** | **Criteria** | **Yes / No** |
| --- | --- | --- |
| 1 | Presence of forefoot diabetic foot osteomyelitis. |  |
| 2 | Age more than or equal to 18 years. |  |
| 3 | Patient willing for a follow-up of 6 months after completion of the antibiotic treatment. |  |
| 4 | Acceptance of local wound care and off-loading. |  |

**1.2 Exclusion Criteria**

| **S. No.** | **Criteria** | **Yes / No** |
| --- | --- | --- |
| 1 | Has the patient refused to give consent to participate in the study? |  |
| 2 | Is the patient pregnant or lactating? |  |
| 3 | Does the patient have peripheral arterial disease? |  |
| 4 | Does the patient have gangrene? |  |
| 5 | Is there an indication for bone resection or amputation? |  |
| 6 | Does the patient have any concomitant infection requiring more than 10 days of systemic antibiotic therapy? |  |
| 7 | Has the patient received more than 5 days of potentially effective systemic antibiotic therapy and is the wound clinically improving? |  |
| 8 | Is there a material-related infection present? |  |
| 9 | Is the patient’s estimated glomerular filtration rate (eGFR) <30? |  |

**Patient ID: __________________ Date of Visit: ____/____/_____**

Informed Consent: ☐ Yes (1) ☐ No (0)

**1.3 Patient Demographics**

| **Parameters** | **Details** |
| --- | --- |
| Serial No: |  |
| Date of presentation: |  |

**1.4 Clinical Data**

| **Parameter** | **Unit** | **Value** |
| --- | --- | --- |
| Age: | Years |  |
| Sex: | Male (1) / Female (2) |  |
| Classification of Diabetes: | Type 1 / Type 2 / Gestational / Other (specify): |  |
| Duration of diabetes: | ____Years & (or) ____ Months |  |
| **Macrovascular Complications** | | |
| Ischemic heart disease | Yes (1) / No (0) |  |
| Cerebrovascular disease | Yes (1) / No (0) |  |
| Peripheral Artery Disease (PAD) | Yes (1) / No (0) |  |
| **Microvascular Complications** | | |
| Diabetic neuropathy | Yes (1) / No (0) |  |
| Diabetic retinopathy | Yes (1) / No (0) |  |
| Diabetic kidney disease | Yes (1) / No (0) |  |

| Hypertension | Yes (1) / No (0) |  |
| --- | --- | --- |
| Dyslipidemia | Yes (1) / No (0) |  |
| Current Smoker | Yes (1) / No (0) |  |

**1.5 History of present illness:**

1. **History of present complaint (Including Duration in Weeks):**

|  |
| --- |
|  |
|  |
|  |

1. **Precipitating factor:**

|  |
| --- |
|  |

1. **Past history of foot complications:**

|  |
| --- |
|  |

1. **Past history of amputation:**

|  |
| --- |
|  |

1. **Treatment history:**

|  |
| --- |
|  |
|  |
|  |

**1.6 General Examination:**

| **Parameter** | **Unit** | **Value** |
| --- | --- | --- |
| Level of consciousness | Glasgow Coma Scale (GCS) |  |
| Weight | kg |  |
| Height (cm): | cm |  |
| BMI: | kg/m² |  |
| Pulse rate: | beats per minute (bpm) |  |
| B.P: | millimeters of mercury (mmHg) |  |
| Respiratory rate: | breaths per minute (breaths/min) |  |
| Temperature: | Degrees Celsius (°C) |  |

**1.7 Examination of the foot:**

| **Parameter** | | | **Right** | **Left** |
| --- | --- | --- | --- | --- |
| Inspection | Evidence of past ulcer | | ☐ Yes (1) ☐ No (0) | ☐ Yes (1) ☐ No (0) |
|  | Previous amputation | | ☐ Yes (1) ☐ No (0)  (Specify: ) | ☐ Yes (1) ☐ No (0)  (Specify: ) |
|  | Ulcer | | ☐ Yes (1) ☐ No (0) | ☐ Yes (1) ☐ No (0) |
|  | Gangrene | | ☐ Yes (1) ☐ No (0)  (Specify: ) | ☐ Yes (1) ☐ No (0)  (Specify: ) |
|  | Foot shape | Prominent metatarsal heads / claw toes | ☐ Yes (1) ☐ No (0) | ☐ Yes (1) ☐ No (0) |
|  |  | Hallux valgus | ☐ Yes (1) ☐ No (0) | ☐ Yes (1) ☐ No (0) |
|  |  | Muscle wasting | ☐ Yes (1) ☐ No (0) | ☐ Yes (1) ☐ No (0) |
|  |  | Charcot deformity | ☐ Yes (1) ☐ No (0) | ☐ Yes (1) ☐ No (0) |
|  | Dermatologic | Callus | ☐ Yes (1) ☐ No (0) | ☐ Yes (1) ☐ No (0) |
|  |  | Erythema | ☐ Yes (1) ☐ No (0) | ☐ Yes (1) ☐ No (0) |
|  |  | Dryness of skin | ☐ Yes (1) ☐ No (0) | ☐ Yes (1) ☐ No (0) |
|  |  | Loss of hair | ☐ Yes (1) ☐ No (0) | ☐ Yes (1) ☐ No (0) |
|  |  | Nails | ☐ Normal (0)  ☐ Thick (1)  ☐ Long (2)  ☐ Ingrown (3)  ☐ Infected (4) | ☐ Normal (0)  ☐ Thick (1)  ☐ Long (2)  ☐ Ingrown (3)  ☐ Infected (4) |
|  |  | Intertrigo | ☐ Yes (1) ☐ No (0) | ☐ Yes (1) ☐ No (0) |
| Neurologic | Motor weakness | | ☐ Yes (1) ☐ No (0) | ☐ Yes (1) ☐ No (0) |
|  | 10-g monofilament test | |  |  |
|  | Vibration sensation using 128 Hz tuning fork | | ☐ Normal (0)  ☐ Impaired (1) | ☐ Normal (0)  ☐ Impaired (1) |
|  | Pinprick sensation | | ☐ Normal (0)  ☐ Impaired (1) | ☐ Normal (0)  ☐ Impaired (1) |
|  | Ankle reflexes | | ☐ Normal (0)  ☐ ↑ (1)  ☐ ↓ (2) | ☐ Normal (0)  ☐ ↑ (1)  ☐ ↓ (2) |
|  | Vibration perception threshold | |  |  |
| Vascular | Foot pulses | | ☐ Normal  ☐ ↓ed (1)  ☐ Not palpable (2)  (Specify: ) | ☐ Normal  ☐ ↓ed (1)  ☐ Not palpable (2)  (Specify: ) |
|  | Ankle Brachial Index | |  |  |
| Ulcer | Location | |  |  |
|  | Size | |  |  |
|  | Grade (University of Texas) | | ☐ 0  ☐ 1  ☐ 2  ☐ 3 | ☐ 0  ☐ 1  ☐ 2  ☐ 3 |
|  | Stage (University of Texas) | | ☐ A  ☐ B  ☐ C  ☐ D | ☐ A  ☐ B  ☐ C  ☐ D |
|  | IWGDF infection severity | | ☐ 3O  ☐ 4O | ☐ 3O  ☐ 4O |

1. **Side of ulcer (Left/Right): ______________________________________________**

**1.8 Investigations**

1. **Hematology / Biochemistry**

| **Parameter** | **Unit** | **Value** |
| --- | --- | --- |
| Hemoglobin | g/dL |  |
| Total count (WBC) | /mm3 |  |
| Neutrophils | /mm3 |  |
| Lymphocytes | /mm3 |  |
| Monocytes | /mm3 |  |
| Eosinophils | /mm3 |  |
| Basophils | /mm3 |  |
| Platelet count | /mm3 |  |
| ESR | mm/hr |  |
| C-reactive protein (CRP) | mg/L |  |

1. **Renal Function Tests (RFT)**

| **Parameter** | **Unit** | **Value** |
| --- | --- | --- |
| Urea | mg/dL |  |
| Creatinine | mg/dL |  |
| Sodium | mmol/L |  |
| Potassium | mmol/L |  |

1. HbA1c: __________________ % Date: ____/____/____
2. **Liver Function Tests (LFT)**

| **Parameter** | **Unit** | **Value** |
| --- | --- | --- |
| Total bilirubin | mg/dL |  |
| Direct bilirubin | mg/dL |  |
| AST (SGOT) | U/L |  |
| ALT (SGPT) | U/L |  |
| ALP | U/L |  |
| GGT | U/L |  |
| Total protein | g/dL |  |
| Albumin | g/dL |  |
| Urine albumin / Creatinine ratio | mg/g |  |

1. **X-Ray Foot (details / impression and date):**

|  |
| --- |
|  |
|  |
|  |

1. **MRI Foot (details / impression and date):**

|  |
| --- |
|  |
|  |
|  |

1. **Arterial Doppler (details / impression and date):**

|  |
| --- |
|  |
|  |
|  |

1. **Culture & Sensitivity - Bone (organism / sensitivity / notes and date):**

|  |
| --- |
|  |
|  |
|  |

1. **Culture & Sensitivity – Tissue, If Available (organism / sensitivity / notes and date):**

|  |
| --- |
|  |
|  |
|  |

1. **Others (Lipid Profile Test)**

| **Parameter** | **Unit** | **Value** |
| --- | --- | --- |
| Total Cholesterol | mg/dL |  |
| Triglycerides | mg/dL |  |
| HDL | mg/dL |  |
| LDL | mg/dL |  |
| VLDL | mg/dL |  |

1. **Others (notes and date):**

|  |
| --- |
|  |
|  |
|  |
|  |

- 1. **Specify (Data Point & Name):**

|  |
| --- |
|  |
|  |
|  |
|  |

**2.0 Visit 1 Checklist:**

| ICF Completed | ☐ Yes |
| --- | --- |
| Inclusion & Exclusion Criteria Verified | ☐ Yes |
| CRF Completely Filled | ☐ Yes |
| All Blood Reports Retained | ☐ Hematology/Biochemistry  ☐ ESR  ☐ CRP  ☐ RFT  ☐ HbA1c  ☐ LFT  ☐ Urine Albumin/Creatinine Ratio  ☐ Lipid Profile |
| Culture & sensitivity Report Retained | ☐ Bone  ☐ Tissue (If Available) ☐ NA |
| Retained Imaging and Other Reports | ☐ MRI Film or CD & Report  ☐ X-Ray Film & Report  ☐ Arterial Doppler Report |
| Ulcer Photograph Captured | ☐ Yes |
| Ulcer Site Marked on Line Diagram | ☐ Yes |
| Principal Investigator (PI) Signature with Date: | ☐ Yes |
|  |  |

**Randomization Sheet**

**Patient ID: __________________ Date of Visit: ____/____/_____**

| **Group Assigned:** | **Antibiotic Advised** | **Drug:** | **Dose:** | **Frequency** |
| --- | --- | --- | --- | --- |
| ☐ 4 Weeks |  |  |  |  |
| ☐ 6 Weeks |  |  |  |  |

**Visit 2 — Day 14**

**Patient ID: __________________ Date of Visit: ____/____/_____**

- 1. **Clinical Assessment:**

1. **Status of Initial Lesion** (Including Dimension, If Healed Mention Date of Healing):

|  |
| --- |
|  |
|  |

1. **New Lesion** (If Any) (Describe: If Ulcer is Present Describe Ulcer Location, Laterality, Size, University of Texas Classification and IDSA Infection Severity):

|  |
| --- |
|  |
|  |

- 1. **Compliance (Medication Adherence)** - ☐ Good ☐ Poor

1. **Compliance Details / Adherence Notes (Expand as Needed):**

|  |
| --- |
|  |

**2.3 Adverse Events** - ☐ Yes ☐ No - If yes, Describe Below

**A. Adverse Event Details (Include Onset, Severity, Action Taken, Outcome):**

|  |
| --- |
|  |
|  |
|  |

- 1. HbA1c, If Available: __________________ % Date: ____/____/____

**2.4 Other Details (Data Point & Name):**

|  |
| --- |
|  |
|  |
|  |
|  |

**2.5 Visit 2 Checklist:**

| Clinical Assessment Completed | ☐ Yes |
| --- | --- |
| Compliance Checked | ☐ Yes |
| Adverse Event Checked | ☐ Yes |
| HbA1c Report Retained (If Available) | ☐ HbA1c ☐ NA |
| Ulcer Photograph Captured | ☐ Yes |
| Ulcer Site Marked on Line Diagram (If New Lesion Identified) | ☐ Yes ☐ NA |
| Principal Investigator (PI) Signature with Date: | ☐ Yes |
|  |  |

**Visit 3 — Day 28**

**Patient ID: __________________ Date of Visit: ____/____/_____**

**3.1 Clinical Assessment:**
**A. Status of Initial Lesion** (Including Dimension, If Healed Mention Date of Healing):

|  |
| --- |
|  |
|  |

1. **New Lesion** (If Any) (Describe: If Ulcer is Present Describe Ulcer Location, Laterality, Size, University of Texas Classification and IDSA Infection Severity):

|  |
| --- |
|  |
|  |

**3.2 Compliance (Medication Adherence)** - ☐ Good ☐ Poor

1. **Compliance Details / Adherence Notes (Expand as Needed):**

|  |
| --- |
|  |

**3.3 Adverse Events** - ☐ Yes ☐ No - If yes, Describe Below

**A. Adverse Event Details (Include Onset, Severity, Action Taken, Outcome):**

|  |
| --- |
|  |
|  |
|  |

**3.4 Hematology / Biochemistry**

| **Parameter** | **Unit** | **Value** |
| --- | --- | --- |
| Hemoglobin | g/dL |  |
| Total count (WBC) | /mm3 |  |
| Neutrophils | /mm3 |  |
| Lymphocytes | /mm3 |  |
| Monocytes | /mm3 |  |
| Eosinophils | /mm3 |  |
| Basophils | /mm3 |  |
| Platelet count | /mm3 |  |

**3.5 Renal Function Tests (RFT)**

| **Parameter** | **Unit** | **Value** |
| --- | --- | --- |
| Urea | mg/dL |  |
| Creatinine | mg/dL |  |
| Sodium | mmol/L |  |
| Potassium | mmol/L |  |

**3.6** HbA1c, If Available: __________________ % Date: ____/____/____

**3.7 Liver Function Tests (LFT)**

| **Parameter** | **Unit** | **Value** |
| --- | --- | --- |
| Total bilirubin | mg/dL |  |
| Direct bilirubin | mg/dL |  |
| AST (SGOT) | U/L |  |
| ALT (SGPT) | U/L |  |
| ALP | U/L |  |
| GGT | U/L |  |
| Total protein | g/dL |  |
| Albumin | g/dL |  |

**3.8 Other Details (Data Point & Name):**

|  |
| --- |
|  |
|  |
|  |
|  |

**3.9 Visit 3 Checklist:**

| Clinical Assessment Completed | ☐ Yes |
| --- | --- |
| Compliance Checked | ☐ Yes |
| Adverse Event Checked | ☐ Yes |
| All Blood Reports Retained | ☐ Hematology/Biochemistry  ☐ RFT  ☐ HbA1c (If Available) ☐ NA  ☐ LFT |
| Ulcer Photograph Captured | ☐ Yes |
| Ulcer Site Marked on Line Diagram  (If New Lesion Identified) | ☐ Yes ☐ NA |
| Principal Investigator (PI) Signature with Date: | ☐ Yes |
|  |  |

**Visit 4 — Day 42 (End of Treatment - EOT)**

**Patient ID: __________________ Date of Visit: ____/____/_____**

**4.1 Clinical Assessment:**

**A. Status of Initial Lesion** (Including Dimension, If Healed Mention Date of Healing):

|  |
| --- |
|  |
|  |

1. **New Lesion** (If Any) (Describe: If Ulcer is Present Describe Ulcer Location, Laterality, Size, University of Texas Classification and IDSA Infection Severity):

|  |
| --- |
|  |
|  |

**4.2 Compliance (Medication Adherence)** - ☐ Good ☐ Poor

1. **Compliance Details / Adherence Notes (Expand as Needed):**

|  |
| --- |
|  |

**4.3 Adverse Events** - ☐ Yes ☐ No - If yes, Describe Below

**A. Adverse Event Details (Include Onset, Severity, Action Taken, Outcome):**

|  |
| --- |
|  |
|  |
|  |

**4.4 Hematology / Biochemistry**

| **Parameter** | **Unit** | **Value** |
| --- | --- | --- |
| Hemoglobin | g/dL |  |
| Total count (WBC) | /mm3 |  |
| Neutrophils | /mm3 |  |
| Lymphocytes | /mm3 |  |
| Monocytes | /mm3 |  |
| Eosinophils | /mm3 |  |
| Basophils | /mm3 |  |
| Platelet count | /mm3 |  |
| ESR | mm/hr |  |
| C-reactive protein (CRP) | mg/L |  |

**4.5 Renal Function Tests (RFT)**

| **Parameter** | **Unit** | **Value** |
| --- | --- | --- |
| Urea | mg/dL |  |
| Creatinine | mg/dL |  |
| Sodium | mmol/L |  |
| Potassium | mmol/L |  |

**4.6** HbA1c, If Available: __________________ % Date: ____/____/____

**4.7 Liver Function Tests (LFT)**

| **Parameter** | **Unit** | **Value** |
| --- | --- | --- |
| Total bilirubin | mg/dL |  |
| Direct bilirubin | mg/dL |  |
| AST (SGOT) | U/L |  |
| ALT (SGPT) | U/L |  |
| ALP | U/L |  |
| GGT | U/L |  |
| Total protein | g/dL |  |
| Albumin | g/dL |  |

- 1. **X-Ray Foot (details / impression and date):**

|  |
| --- |
|  |
|  |
|  |

- 1. **MRI Foot (details / impression and date):**

|  |
| --- |
|  |
|  |
|  |

**5.0 Other Details (Data Point & Name):**

|  |
| --- |
|  |
|  |
|  |
|  |
|  |
|  |
|  |

**5.1 Visit 4 Checklist:**

| Clinical Assessment Completed | ☐ Yes |
| --- | --- |
| Compliance Checked | ☐ Yes |
| Adverse Event Checked | ☐ Yes |
| All Blood Reports Retained | ☐ Hematology/Biochemistry  ☐ ESR  ☐ CRP  ☐ RFT  ☐ HbA1c (If Available) ☐ NA  ☐ LFT |
| Retained Imaging and Other Reports | ☐ MRI Film or CD & Report  ☐ X-Ray Film & Report |
| Ulcer Photograph Captured | ☐ Yes |
| Ulcer Site Marked on Line Diagram  (If New Lesion Identified) | ☐ Yes ☐ NA |
| Principal Investigator (PI) Signature with Date: | ☐ Yes |
|  |  |

**Visit 5 — 2 Months after EOT**

**Patient ID: __________________ Date of Visit: ____/____/_____**
**5.1 Clinical Assessment:**
**A. Status of Initial Lesion** (Including Dimension, If Healed Mention Date of Healing):

|  |
| --- |
|  |
|  |

1. **New Lesion** (If Any) (Describe: If Ulcer is Present Describe Ulcer Location, Laterality, Size, University of Texas Classification and IDSA Infection Severity):

|  |
| --- |
|  |
|  |

**5.2** HbA1c, If Available: __________________ % Date: ____/____/____

**5.3 Other Details (Data Point & Name):**

|  |
| --- |
|  |
|  |
|  |
|  |
|  |
|  |

**5.4 Visit 5 Checklist:**

| Clinical Assessment Completed | ☐ Yes |
| --- | --- |
| Ulcer Photograph Captured | ☐ Yes |
| Ulcer Site Marked on Line Diagram  (If New Lesion Identified) | ☐ Yes ☐ NA |
| HbA1c Report Retained (If Available) | ☐ HbA1c ☐ NA |
| Principal Investigator (PI) Signature with Date: | ☐ Yes |
|  |  |

**Visit 6 — 4 Months after EOT**

**Patient ID: __________________ Date of Visit: ____/____/_____**
**6.1 Clinical Assessment:**
**A. Status of Initial Lesion** (Including Dimension, If Healed Mention Date of Healing):

|  |
| --- |
|  |
|  |

1. **New Lesion** (If Any) (Describe: If Ulcer is Present Describe Ulcer Location, Laterality, Size, University of Texas Classification and IDSA Infection Severity):

|  |
| --- |
|  |
|  |

**6.2** HbA1c, If Available: __________________ % Date: ____/____/____

**6.3 Other Details (Data Point & Name):**

|  |
| --- |
|  |
|  |
|  |
|  |
|  |
|  |

**6.4 Visit 6 Checklist:**

| Clinical Assessment Completed | ☐ Yes |
| --- | --- |
| Ulcer Photograph Captured | ☐ Yes |
| Ulcer Site Marked on Line Diagram  (If New Lesion Identified) | ☐ Yes ☐ NA |
| HbA1c Report Retained (If Available) | ☐ HbA1c ☐ NA |
| Principal Investigator (PI) Signature with Date: | ☐ Yes |
|  |  |

**Visit 7 — 6 Months after EOT**

**Patient ID: __________________ Date of Visit: ____/____/_____**

**7.1 Clinical Assessment:**
**A. Status of Initial Lesion** (Including Dimension, If Healed Mention Date of Healing):

|  |
| --- |
|  |
|  |

1. **New Lesion** (If Any) (Describe: If Ulcer is Present Describe Ulcer Location, Laterality, Size, University of Texas Classification and IDSA Infection Severity):

|  |
| --- |
|  |
|  |

**7.2 Investigations:**

| **Parameter** | **Unit** | **Value** |
| --- | --- | --- |
| ESR | mm/hr |  |
| C-reactive protein (CRP) | mg/L |  |

**7.3** HbA1c, If Available: __________________ % Date: ____/____/____

**7.4 Medical History:**

|  |
| --- |
|  |
|  |

**7.5 MRI Foot (details / impression and date):**

|  |
| --- |
|  |
|  |
|  |

**7.6 X-Ray Foot (details / impression and date):**

|  |
| --- |
|  |
|  |
|  |

**7.7 Other Details (Data Point & Name):**

|  |
| --- |
|  |
|  |
|  |
|  |

**7.8 Visit 7 Checklist:**

| Clinical Assessment Completed | ☐ Yes |
| --- | --- |
| All Blood Reports Retained | ☐ ESR  ☐ CRP  ☐ HbA1c (If Available) ☐ NA |
| Retained Imaging and Other Reports | ☐ MRI Film or CD & Report  ☐ X-Ray Film & Report |
| Ulcer Photograph Captured | ☐ Yes |
| Ulcer Site Marked on Line Diagram  (If New Lesion Identified) | ☐ Yes ☐ NA |
| Principal Investigator (PI) Signature with Date: | ☐ Yes |
|  |  |

**FINAL OUTCOME VISIT**

**Patient ID: __________________ Date of Visit: ____/____/_____**

| Initial Ulcer | ☐ Healed (1) ☐ Not Healed (0)  If Healed,  Healing Time (In Weeks):  Date of Healing: ____/____/____  If Not Healed, Details: |
| --- | --- |
| Amputation / Surgical Resection | ☐ Yes (1) ☐ No (0)  If Yes, specify: |
| Relapse | ☐ Yes (1) ☐ No (0)  Date of Relapse: ____/____/____ |
| Remission | ☐ Yes (1) ☐ No (0) |
| Death | ☐ Yes (1) ☐ No (0) |
| Drop out | ☐ Yes (1) ☐ No (0)  If Yes, Reason: |
| Investigator Signature / Date |  |

**Visit -** ______**
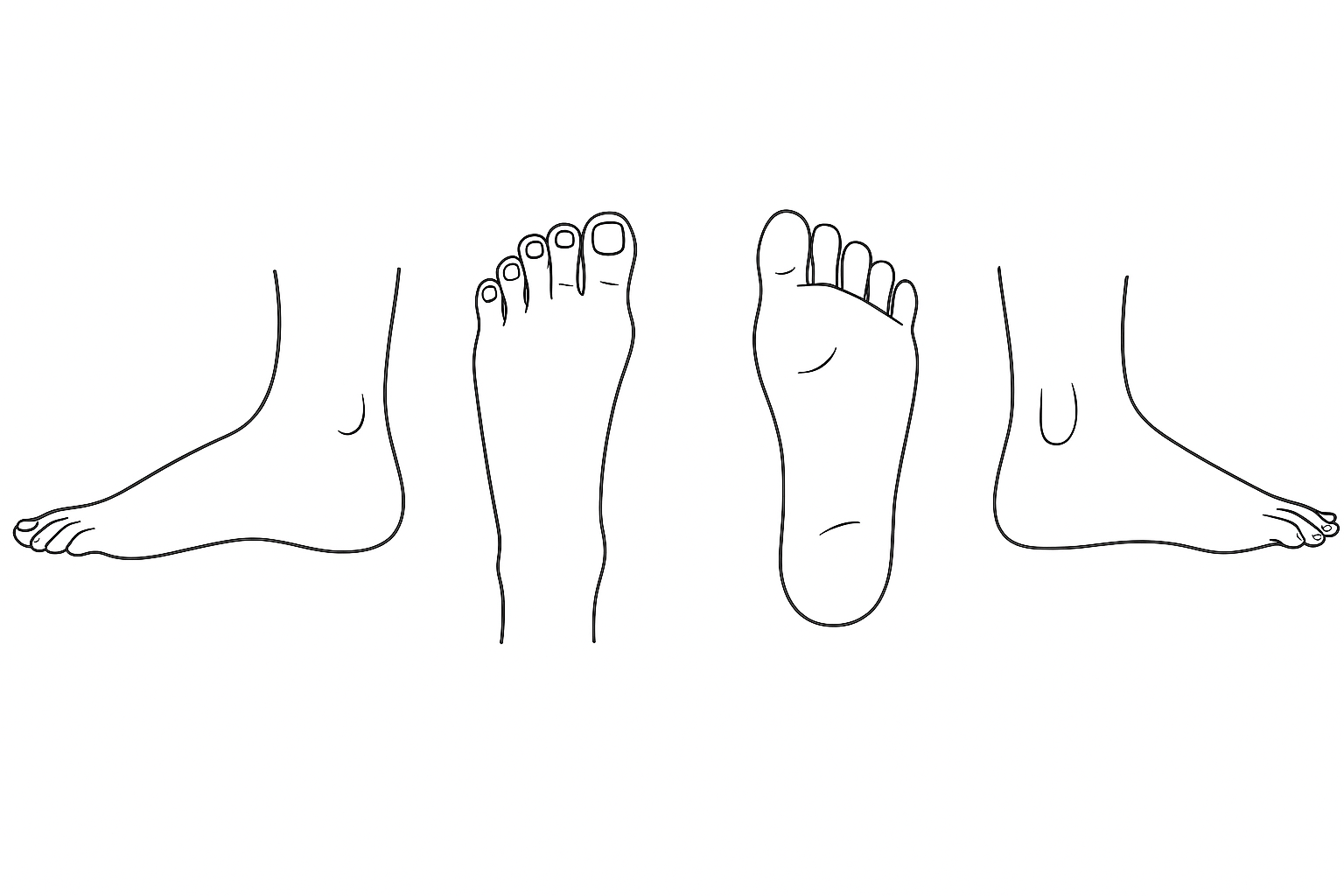
**

**Ulcer Location:** ☐ Left Foot ☐ Right Foot,

**Unscheduled Visit**

**Patient ID: __________________ Date of Visit: ____/____/_____**

**U.1 Reason for Unscheduled Visit:**

|  |
| --- |
|  |
|  |

**U.2 Clinical Assessment:**
**A. Status of Initial Lesion** (Including Dimension, If Healed Mention Date of Healing):

|  |
| --- |
|  |
|  |

1. **New Lesion** (If Any) (Describe: If Ulcer is Present Describe Ulcer Location, Laterality, Size, University of Texas Classification and IDSA Infection Severity):

|  |
| --- |
|  |
|  |

**U.3 Add Parameters as Required:**

|  |
| --- |
|  |
|  |
|  |
|  |
|  |
|  |

**Additional Notes Sheet:**

**Patient ID: __________________ Visit: ____ Data Point: __________**

**Notes (Expand as Needed):**

|  |
| --- |
|  |
|  |
|  |
|  |
|  |
|  |
